# The Varieties, Causation, and Treatment of Appendicitis

**Published:** 1908-10-03

**Authors:** George Thos. Beatson

**Affiliations:** Surgeon to the Glasgow Western Infirmary, and to the Glasgow Cancer Hospital


### HOSPITAL CLINICS.

### THE VARIETIES, CAUSATION, AND TREATMENT OF APPENDICITIS.

By SIR GEORGE THOS. BEATSON, K.C.B., M.D., Surgeon to the Glasgow Western Infirmary, and to the Glasgow Cancer Hospital.

I HAVE chosen as the subject of my lecture to-day the varieties, causation, and treatment of appendicitis, because you have seen operated on this week, on consecutive days, three cases of this affection, all differing from one another, but serving as an instructive text for my remarks. I admit the term "appendicitis," with its Latin root and its Greek termination, may not be very correct etymologically, but it is one that is now so generally employed that it would be useless to try to displace it, especially as it indicates what has been clearly established—that the appendix is the origin of most of the inflammatory attacks that occur in the right iliac region. At the same time, it must be remembered that the appendicitis of to-day is not a new disease, but is the modern name for the older affections known as typhlitis, perityphlitis, iliac abscess, and iliac phlegmon.

Structurally the appendix wall has the same composition as that of the colon, and its mucous membrane is abundantly supplied with the same glands as are found in the cæcum. So numerous are these glands, and so closely packed together, that the mucous membrane of the appendix is quite lymphoid in its character. Anatomically the average length of the appendix is 4½ inches, and its normal diameter is said to be that of a No. 9 English catheter; but this is not so all through life, as the organ is the seat of a physiological atrophy (Ribbert), especially after the thirtieth year. The appendix is attached to the cæcum behind, and to its inner side, but its root or origin is not constant, and it may be more to the right or left. Speaking generally, it may be said that the cæcal orifice of the appendix is situated on the postero-internal aspect of the cæcum, and does not correspond exactly with McBurney's point, where, as you know, the chief pain is felt on palpation in acute appendicitis. At the root of the appendix is a semi-circular fold of mucous membrane, known as the valve of Gerlach, which practically separates its lumen from that of the cæcum, and prevents the passage of fæcal matter into the appendix. The nerves of the appendix are derived from the mesenteric plexus of the sympathetic, and its blood-supply is furnished by an artery which springs from the superior mesenteric, and feeds the appendical wall by means of anastomosing branches. In woman there is an additional blood-supply from a branch of the ovarian artery which runs in the appendicular-ovarian ligament. We shall see subsequently that the question of the blood-supply of the appendix is an important one, as interference with it is a fertile cause of disease in the organ. Lastly, I would draw special attention to another very important factor in connection with appendicitis, and that is the relation of the appendix to the general peritoneal cavity. As a general rule, it is in direct communication with it, being usually covered with peritoneum and possessing a small mesentery of its own. At the same time, this is not the invariable state of matters, and in a certain percentage of cases the appendix has no serous coat, but occupies a "retro-peritoneal" position in the cellular tissue behind the cæcum.

Passing now to the consideration of the appendix pathologically, this fact stands out clear and distinct, that it is very liable to inflammation, especially between ten and forty, the frequency decreasing with age, so that after the fortieth year the disease is not so common. The form the inflammation may take varies, and may be (1) catarrhal, (2) ulcerative, or (3) gangrenous. In the same way it may have its seat in the (1) mucous membrane, (2) the appendicular wall, (3) the serous covering, or (4) in all these three structures combined.

It is when the serous coat is attacked that inflammation of the appendix becomes a serious matter, for we are then face to face with peritonitis. It is this latter that has to be treated, and the case can only be satisfactorily and successfully carried through by bearing this in mind. Further, it is only by an exact knowledge of what the course of events may be in a case of appendicular peritonitis that a correct decision can be come to in the management of a case. Recent years have furnished a great deal of valuable information on this point, owing to the vast amount of operative work that has been done in connection with acute and chronic cases of this affection, and I think the outcome of the experience of all surgeons is that inflammation of the appendix manifests itself in three distinct varieties, which I would designate as (1) serous, (2) fibrinous, (3) suppurative.

In serous appendicitis peritonitis shows itself by mere congestion of the surface of the appendix, with or without adhesions. When these latter are present they are generally of a gauzy filmy kind, and may be either local or widespread. On the other hand, in fibrinous appendicitis we find the appendix and the adjacent tissues coated with thick fibrinous lymph, which may be limited to the cæcum, or may be very generally disseminated through the coils of the intestine. Lastly, in suppurative appendicitis, we have a purulent secretion which bathes the appendix, and forms a collection of pus around it, a state of matters which may be confined to the right iliac region, or may involve the whole peritoneal cavity. This additional and very important fact should never be lost sight of, that such a peritonitis may be local, or may be general, the former being by far the more common.

Let me now briefly point out what are the usual terminations of the above varieties of acute appendicitis; in other words, what are the morbid conditions we may have to treat. It will be found, clinically, that the cases vary with the variety of inflammation present. A serous peritonitis is usually local, and yields readily to the medical measures I shall speak of later on. It leaves, perhaps, no traces behind it, save possibly some adhesions. The same satisfactory result occurs in the cases of fibrinous peritonitis. They are of longer duration, and adhesions of a dense and fibrous character result, but they terminate successfully as a rule under medical treatment.

In suppurative appendicitis the pus may be local, or it may attack the whole peritoneal cavity. When the abscess is local it may be of small size, and completely circumscribed by matted intestines or omentum, or it may be of considerable dimensions and similarly shut in. In both cases it is intra-peritoneal. Should the appendix be retro-peritoneal, or should the abscess perforate the iliac fascia, then the pus is generally large in amount, and is extra-peritoneal. As to the ultimate course of these local collections of pus, it is important to have a clear understanding. Those of small size are, I am satisfied, often absorbed, leaving behind them that yellowish ochre-coloured debris that one often finds around the appendix when operating on cases in a quiescent stage. On the other hand, abscesses, both small and large, may make their way outwards and burst externally, or they may open into the bowel, or into the general peritoneal cavity. As to which of these routes an abscess will take, it is impossible to say positively, but an idea is given by studying groups of cases. Thus, in sixty-seven collected by Bull, it was found that thirty-eight opened externally through the abdominal wall, fifteen entered the bowel, eight the general peritoneal cavity, and two each of the remaining six made their way respectively into the thorax, bladder, and rectum. I wish you specially to note these figures, as the fear of these abscesses bursting into the peritoneum is put forward as an argument in favour of that early operative interference in suppurative appendicitis that is responsible, I consider, for the high mortality that accompanies that line of treatment. My own experience is that with proper management rupture of an appendicular abscess into the general peritoneal cavity is a very unlikely occurrence, and is not borne out by clinical facts.

I do not know that there are any very definite statistics indicating clearly the proportion of cases with localised and with diffuse suppuration, but I think the latter are in a small minority. Hawkins' figures, published from St. Thomas's Hospital in 1895, give 36 cases of general peritonitis out of 264, but in 250 cases under my own care, the number is very much lower than 36. As regards the issue in these cases of suppurative appendicular peritonitis, those with localised suppuration, if judiciously handled, do well; but the mortality in general peritonitis is very high. It is this that is responsible for a fatal issue, and it is the prevention of this that should form the basis of all our therapeutic measures.

Let me now briefly draw your attention to another point about cases of appendicitis that you should bear in mind. Although a large number of them recover from their first attack without operation—an encouraging fact that is perhaps not sufficiently recognised—there is, in a considerable proportion of them, a liability to the affection becoming chronic, or to its recurrence after a varying period of apparently sound health. To this latter condition has been given the name of relapsing or recurring appendicitis. Fitz gives the frequency of this recurrence as 44, and Hawkins as 23 per cent., but the former figures are probably the correct ones. When we come to consider the causation of appendicitis and see how twists and bends of the appendix, as well as partial stenosis of its lumen, leading to accumulation of its fluid contents, are important factors in the causation of the disease, it is not difficult to understand the occurrence of these recurring attacks.

Looking to the varying conditions that I have just described, I consider the following is the best classification of cases of appendicitis, or, as I would prefer to call them, appendicular peritonitis: (1) Acute appendicitis with localised peritonitis, either (*a*) serous, (*b*) fibrinous, or (*c*) suppurative. (2) Acute appendicitis with general peritonitis, invariably suppurative. (3) Chronic appendicitis. (4) Relapsing appendicitis, marked by acute or sub-acute exacerbations of a serous, fibrinous or suppurative character, but with periods of sound health between.

The causes that set up the disease, in their order of frequency, I regard as: (1) Bacterial infection, usually by the pus cocci and the bacterium coli. (2) Irregular closures of the lumen of the appendix, causing retained secretions. (3) Twists and bends of the appendix and its mesentery. (4) Foreign bodies, including concretions.

In giving bacteria the foremost place in the causation of the inflammation of the appendix, it must be understood that they need some favouring factor to allow them to come into play, for pus cocci and the bacteria coli are regularly found in normal appendices. Observation and experiment seem to show that what is required is that they must exist under pressure. Unless this is present, they cannot excite inflammation, a proposition that holds equally good for skin, mucous membrane, or surface wounds. In the case of the appendix, there are many conditions that set up this increased pressure. For instance, within the organ, we have swelling of the mucous membrane of the valve of Gerlach and irregular obliterations of its lumen. Both these shut in the secretions and increase the tension. The same thing happens as the result of those external bends and twists of the appendix and its mesentery, which materially interfere with its blood-supply and modify the circulation through its tissues. Foreign bodies and concretions act in the same way, that is by increasing the vascularity, and in connection with both of these, as well as with retained secretions, ulceration is apt to occur, for all of them by their pressure erode the tissues until they give way. When this happens, it is not difficult to understand how the sudden escape of a septic fluid, or of a foreign body or concretion, into the peritoneal cavity sets up a general peritonitis. Other assigned causes of appendicitis are blows, falls, excessive physical exertion, fatigue, improper food and digestive disturbances, exposure to cold, rheumatism, influenza, and typhoid fever; but they must rather be looked upon as pre-disposing causes, that favour the action of the micro-organisms present.

As you have seen a good many cases of appendicitis admitted during the session, I need not dwell on the symptoms of the disease. You will remember that we have had different clinical varieties of it. The (1) chronic, the (2) relapsing, and (3) the acute. The chronic cases had a history of an acute attack, which subsided, but left a more or less indefinite uneasiness in the right iliac region, and disturbed digestion. Examination did or did not reveal any local hardness or thickening, but very often tenderness on deep pressure at McBurney's point. Relapsing cases that were admitted for operation in a healthy interval were devoid of symptoms, and reliance had to be placed on the history given by the patients, supplemented, when it is possible, by the opinion of the medical man who had attended during the illness. When, however, they were admitted during an acute exacerbation, they presented the symptoms that marked the cases of acute appendicitis suffering from a first attack. These, you will remember, were as follows: (1) Pain in the abdomen, very often of sudden onset, and referred at first to the epigastrium, but eventually to the right iliac fossa; (2) constitutional disturbance as shown by quick pulse and fever of varying degree; (3) gastro-intestinal disturbance, manifesting itself by nausea, vomiting, and usually constipation; (4) localised symptoms in the right iliac region—namely, tenderness over the root of the appendix (McBurney's point), muscular rigidity, amounting sometimes to hardness, and dulness. In addition the posture of the patient was often very characteristic, the dorsal position being assumed with the right leg flexed on the abdomen.

With such a group of symptoms present in a young person, the diagnosis should be that of acute inflammation around the appendix. At the same time there are other conditions, such as biliary and renal colic, ruptured gastric ulcer, tubal and ovarian disease in women, with which it might be confounded.

The diagnosis of appendicitis having been made, there has then to be settled the very important question of the treatment of the case. No difficulty presents itself when it is one of the chronic or relapsing variety, in a quiescent stage. Provided the patient is otherwise healthy, removal of the appendix is the proper treatment. The experience of all surgeons—and it is mine also—is that the mortality in these cases is very small. During the last ten years at this hospital I have removed the appendix in nearly 150 interval cases, when the acute symptoms have subsided, and I have not lost one.

It is in cases of acute appendicitis, with its accompanying peritonitis, that the line of treatment to be followed is fraught with so much of good or ill for the patient. The first point that has to be settled is whether or not the peritonitis present is (1) general or (2) local.

In the treatment of acute general peritonitis the peritoneal cavity should be opened, preferably by a median incision low down, its interior rapidly examined by the finger for any accumulation of pus in the region of the appendix, and drainage established by one or more good-sized drainage tubes. No time should be occupied in flushing or wiping out the interior of the abdomen. Rapid laparotomy with drainage is preferable to flushing. As it has been tersely put, the rule in these cases should be "Quick in and quick out." The patient should then be put to bed in the semi-recumbent or Fowler position, and the method of treating diffuse septic peritonitis advocated by Dr. Le Conte of Philadelphia, by means of introducing large quantities of fluid into the rectum, commenced. I will not go at length into the details of the method, because you have seen it carried out, and the procedure was explained to you. I would remind you that no special apparatus was employed. What was used was a catheter with tubing, and a Higginson's syringe, care being taken that the fluid had a temperature of about 100° F., and that it was introduced at the rate of about a pint an hour.

While, however, placing our main reliance on this continuous rectal injection of fluid, other measures must not be neglected. Strychnine and digitaline should be given hypodermically to combat the cardiac depression induced by the toxic element in these cases. Stimulants, too, may be necessary, and easily assimilated nourishment should be given in the form of albumen water or chicken tea. Should there be much gastro-intestinal disturbance as shown by vomiting and flatulent distension, a powder of bismuth, soda, and grey powder should be administered every four hours, and to it there may be added with advantage a small quantity of Dover's powder, according to age, should there be pain or restlessness to any marked degree. As warmth to the abdomen in my opinion favours that local leucocytosis which is so helpful in these cases, hot boric fomentations should be employed as a dressing over the drainage tube, and changed every few hours.

Fortunately cases of general peritonitis form a small percentage in acute inflammation of the appendix, and we are usually called upon to treat a localised peritonitis. I admit it is not always easy to realise this fact, for a great deal of constitutional disturbance and very alarming abdominal symptoms are apt to be set up by a very limited appendicular inflammation. But it is very important not to be misled by this state of matters, and one must rely on the presence or absence of that general rigidity with hyperæsthesia of the abdomen, that rapid pulse and moderate temperature, that characteristic facial expression that point to general peritonitis. Considerable clinical experience of appendicitis has convinced me that to follow the rule that has been laid down by some, that acute and violent symptoms call for abdominal section, is an error, and often a disastrous one. In fact, my experience in both hospital and private work is that early operative measures in acute appendicitis with localised peritonitis are not called for, save to the very limited extent of opening an abscess that may be pointing. The fact is not sufficiently recognised that the great majority of cases of acute localised inflammation of the appendix, be it serous, fibrinous, or suppurative, may be kept localised and subdued by suitable treatment, thus allowing of their being safely operated on when the acute condition has subsided. I am aware that the teaching is that operation should be done as soon as suppuration is established, or if gangrene or perforation is suspected. From this teaching I dissent. I hold strongly that unless general peritonitis is present these conditions should make one hesitate to operate. As long as they are localised, and fortunately they generally are, no immediate danger to life exists, while the chances of causing the general peritonitis we so much dread are very great if any operative measures are adopted. No matter what care be taken, it is almost impossible to avoid its occurrence, and the heavy mortality that marks the surgical treatment of acute appendicitis on these lines is mainly due to what I may call operative general peritonitis. In my opinion, a localised intra-peritoneal suppuration around the appendix is best left alone in its acute stage, and that for several reasons.

In the first place, the acuteness of the attack may be subdued by medical treatment, and the pus, if small in amount, absorbed. Secondly, if not, such collections of pus are given an opportunity of adhering to the parietal peritoneum when they come to the outside and are safely opened. McGuire's statistics give twenty-three adherent abscesses opened without a death, while of seventy non-adherent cases so treated there were seven deaths. I know that this waiting treatment does not find general acceptance, and there is at once raised the cry that it is almost criminal to leave such cases, as one never knows the day when the pus will burst into the general peritoneal cavity and prove fatal. The fact is, that experience shows that it does not do so, if proper medical general treatment is carried out on the lines I shall give you later on. I have followed this waiting method during ten years of hospital practice, and I have had no fatal cases following it. What has happened has been that those cases that have not been absorbed or have burst externally, have opened into the cæcum or rectum. Some half-dozen cases have done so, and the results have been quite satisfactory. All this has strengthened me in adherence to the rule of non-interference in the early stage of acute localised suppuration, for I am satisfied that this dread of rupture into the peritoneum is an unfounded one; that it is illogical and unfair to patients to follow what cannot but be regarded as a risky line of treatment, based on the possible occurrence of a grave condition which may not happen, and has been shown by experience to be comparatively rare.

Surgeons who would hesitate to open an abdomen if they have recently handled a purulent septic case will cut down without hesitation on a limited collection of actively virulent pus inside the peritoneum, and going through the necessary manipulations on the threshold of that still uninvaded cavity, will expect not to infect it. A moment's reflection will show the great danger that is run by doing so—in my opinion far out of proportion to that involved by the waiting policy. Further, this latter course has the advantage that with time the collection of pus loses its virulence, as I have verified by culturing the pus from such abscesses. Operative measures are consequently safer when this is so. My rule in acute cases is to subdue the symptoms, and to wait until pulse, temperature, and blood-count have remained normal for some days before operating. If the leucocytosis remains high after pulse and temperature have fallen, then I delay operation, but not indefinitely, for I know that with a lapse of two or three weeks the abscess is possibly adherent to the parietal peritoneum, and certainly the pus has become less virulent. When operation is decided on, the removal of the appendix will depend on the condition found. If a distinct abscess is present, it is better to drain it and let it heal, before dealing with the appendix itself.

Let me now briefly recapitulate the medical measures that I advocate and lay great stress on in acute appendicitis. They are all based on the principle of preventing peristalsis of the intestinal canal. It is the movement of the intestines that is the great hindrance to the localisation of an appendicular inflammation, while it is also the main factor in disseminating sceptic material through the peritoneal cavity. Hence it is essential that the intestines be kept at rest. This can only be done by opium, and the preparation I use is Dover's powder in proportion to the age of the patient. Adults have 5 grains or $2\frac{1}{2}$ grains according to the amount of pain, and children in proportion. It is repeated every two, three, and four hours according to circumstances. The argument that opium is to be withheld because it masks symptoms cannot be entertained for one moment, when it is essential to arrest those peristaltic movements of the intestines which help above all to spread in the peritoneal cavity the deleterious products of acute septic inflammation.

On the same ground, Lawson Tait's treatment by saline purges is entirely out of place. I am satisfied that it is the neglect of opium, and the use of saline purges, that are responsible for the unsatisfactory progress of many cases that would otherwise have had a favourable course. Along with the Dover's powder, I combine an equal quantity of subnitrate of bismuth, and in cases with much gastro-intestinal disturbance, as shown by sickness or flatulence, I add a small dose of grey powder ($\frac{1}{4}$ grain) to each powder, and employ the rectal tube to carry off flatus. The grey powder is not given for any laxative effect, but as a most useful intestinal antiseptic. In fact, I never use laxatives in these cases. I have the large intestine emptied by soap and water enemata, but I do not aim at any general lavage of the colon.

Locally, I use warmth in the shape of light and hot poultices. The food administered is restricted to small quantities of albumen water, which is nourishing, and sets up no peristalsis of the intestine, as it is absorbed by the stomach. The patient is placed in the position of the greatest comfort, but in the Fowler one if there is much flatulence, or any hiccough. I continue treatment on these same lines until the acute symptoms yield, for once having adopted it I put away all idea of surgical interference until the attack is over, unless conditions —such as the pointing of an abscess, or clear indications of general suppurative peritonitis—distinctly call for it.

Ten years of hospital work have fully strengthened me in the above line of treatment, and I am more and more convinced that operative treatment in the early stages of acute appendicitis is called for in a very small percentage of cases, and that the majority of them can be more safely carried through the attack by medical measures, than dealt with surgically. At the Western Infirmary I have treated during the past ten years 161 cases of appendicitis, with six deaths. This gives a mortality of 3.7 per cent. I have reason to know that my line of treatment is at variance with the teaching of some of my colleagues, but I am satisfied this question of the treatment of acute appendicitis is not the purely surgical one that is so generally assumed.

